# Diverse and nonlinear influences of built environment factors on COVID-19 spread across townships in China at its initial stage

**DOI:** 10.1038/s41598-021-91849-1

**Published:** 2021-06-14

**Authors:** Shuang Ma, Shuangjin Li, Junyi Zhang

**Affiliations:** 1 Research Center for Advanced Science and Technology, The University of Tokyo, Tokyo, 153-8904 Japan; 2 Graduate School for International Development and Cooperation, Hiroshima University, Higashi Hiroshima, 739-8529 Japan; 3 Graduate School of Advanced Science and Engineering, Graduate School for International Development and Cooperation, Hiroshima University, Higashi Hiroshima, 739-8529 Japan

**Keywords:** Infectious diseases, Environmental social sciences

## Abstract

The built environment can contribute to the spread of the novel coronavirus disease (COVID-19) by facilitating human mobility and social contacts between infected and uninfected individuals. However, mobility data capturing detailed interpersonal transmission at a large scale are not available. In this study, we aimed to objectively assess the influence of key built environment factors, which create spaces for activities—“inferred activity” rather than “actually observed activity”—on the spread of COVID-19 across townships in China at its initial stage through a random forest approach. Taking data for 2994 township-level administrative units, the spread is measured by two indicators: the ratio of cumulative infection cases (RCIC), and the coefficient of variation of infection cases (CVIC) that reflects the policy effect in the initial stage of the spread. Accordingly, we selected 19 explanatory variables covering built environment factors (urban facilities, land use, and transportation infrastructure), the level of nighttime activities, and the inter-city population flow (from Hubei Province). We investigated the spatial agglomerations based on an analysis of bivariate local indicators of spatial association between RCIC and CVIC. We found spatial agglomeration (or positive spatial autocorrelations) of RCIC and CVIC in about 20% of all townships under study. The density of convenience shops, supermarkets and shopping malls (DoCSS), and the inter-city population flow (from Hubei Province) are the two most important variables to explain RCIC, while the population flow is the most important factor in measuring policy effects (CVIC). When the DoCSS gets to 21/km^2^, the density of comprehensive hospitals to 0.7/km^2^, the density of road intersections to 72/km^2^, and the density of gyms and sports centers to 2/km^2^, their impacts on RCIC reach their maximum and remain constant with further increases in the density values. Stricter policy measures should be taken at townships with a density of colleges and universities higher than 0.5/km^2^ or a density of comprehensive hospitals higher than 0.25/km^2^ in order to effectively control the spread of COVID-19.

## Introduction

Infectious diseases are usually caused by face-to-face interpersonal communications or contacts^1–3^. Capturing such interpersonal communications or contacts at various locations over time at a large scale is useful to better understand and consequently control the spread of infectious diseases. This argument is also applicable to the spread of the novel coronavirus disease (COVID-19). However, in reality, such micro-level data is not available at a large scale, making it necessary to make use of other indicators as a proxy for interpersonal communications or contacts. In some cases, telephone-based or online questionnaire surveys have been implemented to explore face-to-face communications in the built environment^4^. For instance, Zhang et al.^5^ implemented a telephone-based survey of 1021 persons in Hong Kong to explore the changes in close contact rates under different indoor environments such as workplaces, restaurants, and shopping centers. However, it is too costly to collect sufficiently large-scale samples that can represent the whole population. A few studies have investigated the large-scale inter/intra-city contacts on public transportation through smart card data of railways^6^ and flight information^7^, but such data cannot reflect human mobility in various urban facilities.

This study focuses on the roles of the built environment in explaining the spread of COVID-19 at its initial stage in China. As stated by Roof and Oleru^8^, the built environment can be defined as "the human-made space in which people live, work and recreate on a day-to-day basis." In other words, it provides the setting for human activities. Built environment factors can therefore be used to indirectly represent human mobility over time and across space. The built environment may alter the spread of COVID-19 by facilitating human mobility and social contacts between infected and susceptible individuals. It can also directly introduce pathogens to the susceptible individuals.

Most existing studies which investigate the transmission of communicable diseases in association with the built environment focus on single factors such as population distributions and mobility across space^5,9,10^. Physical distance is often used to predict the disease propagation. For example, scholars have investigated the critical immunization radius based on physical distance among people in small-world networks^11^. The distance between different administrative districts was computed to evaluate visitor volumes using a gravity model^12^. The epidemic dynamics of the transmission were studied by evaluating the commuting connections based on a metapopulation model^13^. The predictability and reliability of the pandemic forecast were examined using the international flow of flights^14^. Several scholars also applied a pedestrian path to evaluate disease transmission. For example, pedestrian paths were used to evaluate the severe acute respiratory syndrome (SARS) transmission^15^. The real-time synthesis of crowd motion was investigated for thousands of individuals with intersecting paths^16^. However, it has remained unclear how different built environment factors may jointly contribute to the spread of communicable diseases, limiting the scientific evidence for designing effective policies.

Given the need for more evidence regarding the built environment to help control the spread of COVID-19, we conducted a nationwide study in China at its initial stage of the COVID-19 pandemic. Specifically, we first delineated the spatial agglomeration of the two dependent variables, i.e., the ratio of cumulative infection cases (RCIC), and the coefficient of variation of infection cases (CVIC), based on an analysis of bivariate local indicators of spatial association (BiLISA). Then, we objectively investigated the diverse and nonlinear influences of 19 explanatory variables on the spread of COVID-19, including built environment factors (related to urban facilities, land use, and transportation infrastructure), the levels of nighttime activities (measured by nighttime light data), and the inter-city population flow (from Hubei Province), based on a random forest approach. Finally, we discussed the policy implications of our findings. Significant contributions of this study are (1) to clarify various nonlinear influences of built environment factors on the spread of COVID-19 in China at the township level, and (2) to identify thresholds of built environment factors that are useful to guide future urban planning against pandemics.

## Results

### Spatial agglomeration of the RCIC and CVIC

**Figure 1:**
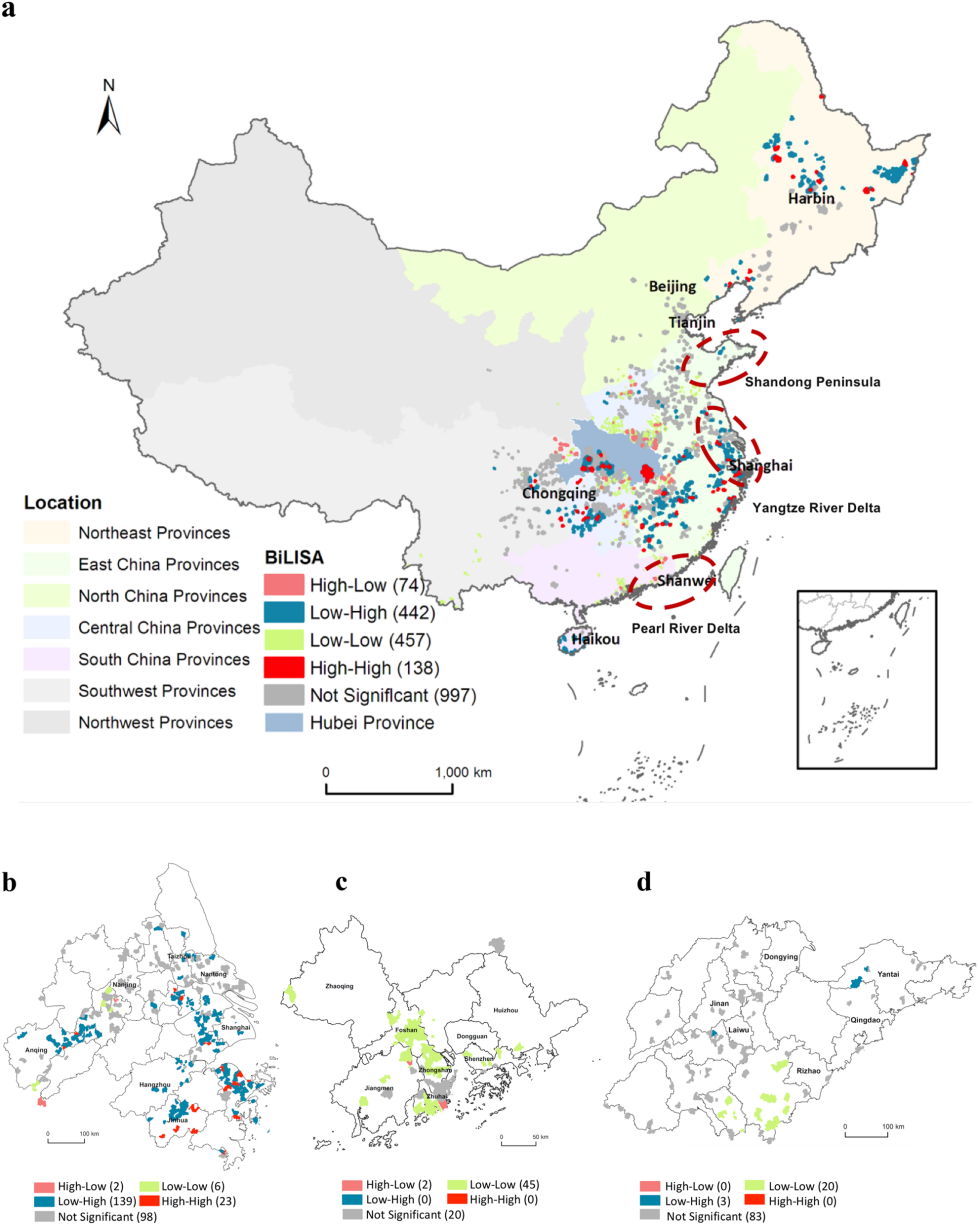
The BiLISA map showing the spatial distribution of the RCIC and CVIC. (**a**) Whole of China. (**b**) Yangtze River Delta. (**c**) Pearl River Delta. (**d**) Shandong Peninsula. Created using ArcMap Version 10.2 from ESRI (http://www.arcgis.com/).

The BiLISA-based method delineates a spatial agglomeration of the RCIC and CVIC, as seen in Fig. 1. By focusing on 2108 townships with infection cases (out of the total 2994 townships), we found positive autocorrelations in 595 (138 High–High types and 457 Low–Low types). The High–High type (6.5% out of the 2108 townships) represents a cluster with high RCIC accompanied by neighboring townships with less effective policies, as indicated by high CVIC (denoted by red color). This type is mainly situated in Chongqing, the provinces in Central China, and the provinces in northeastern China. This finding means that more infections in the above clusters may be due to less effective policies made at their neighboring townships. The Low–Low type (21.7%) indicates that a cluster with low RCIC is observed together with a cluster with low CVIC (denoted by green color), and it is mainly distributed in the provinces in Central China; specifically, those townships around Hubei Province, the southeast provinces, and southwest provinces. This type is mostly observed in the whole China, implying that infections at most of the townships in China are associated with effective policies made at their neighboring townships. In other words, collective efforts are crucial to control the spread of COVID-19 virus. The negative autocorrelations (outliers) are observed between the RCIC and CVIC in the High–Low type (denoted by pink color) and the Low–High type (denoted by blue color), which are found in 74 and 442 townships, respectively. The High–Low type (3.5%) is mainly detected at townships surrounding Hubei Province, meaning that infections at these townships, close to the virus center, are less likely to be affected by effective policies at their neighboring townships. The Low–High type accounts for 21.0% out of the 2108 townships with infection cases. This share is similar to that of the Low–low type. This means that the virus spread at more than one-fifth of the townships with infection cases may be controlled by their own efforts, even though policies made at their neighboring townships were less effective. This finding further suggests that successful policies need to be better transferred across neighboring townships.

Based on the spatial agglomerations in three typical city groups (Fig. 1b,c) in the Yangtze River Delta city group, positive autocorrelations are found in 17.1% of the townships, within which 79.3% are High–High types and 20.7% are Low–Low types. In the Yangtze River Delta city group, the Low–High type has the largest distribution with 81.8%. In the Pearl River Delta city group, all townships belong to the Low–Low type. In the Shandong Peninsula urban agglomeration, 87.0% of its townships have positive autocorrelations in the Low–Low type, and the remaining percentage is observed with negative autocorrelations (Low–High type).

### Impacts of the built environment indicators on the spread of COVID-19

**Figure 2:**
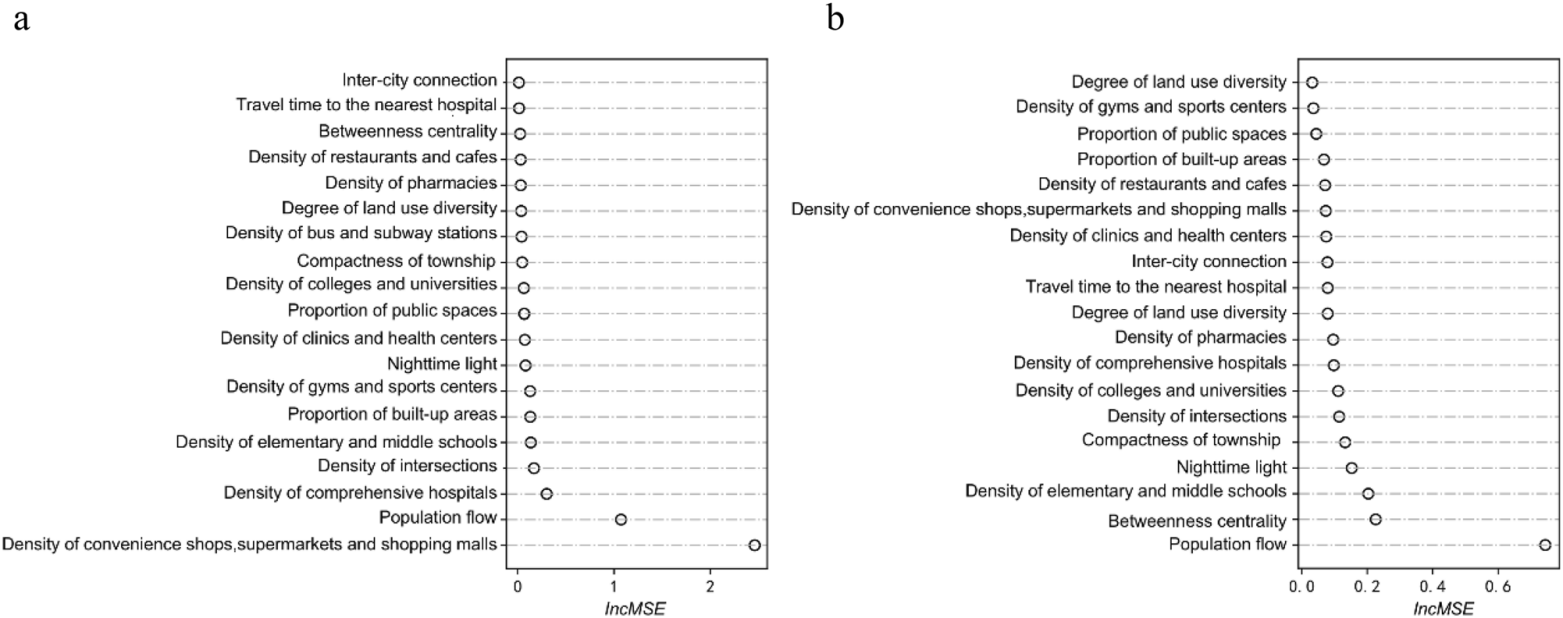
Importance of built environment factors in the spread of COVID-19. (**a**) Relative importance of built environment factors to RCIC. (**b**) Relative importance of built environment factors to CVIC. Created using Anaconda Navigator Version 1.9.12 from Anaconda (https://www.anaconda.com).

Figure 2 illustrates the relative importance [measured by increased mean square error (IncMSE)] of all 19 explanatory variables to RCIC (Fig. 2a) and CVIC (Fig. 2b), derived from the random forest approach. The density of convenience shops, supermarkets and shopping malls (DoCSS) and the inter-city population flow are the two most important variables for the RCIC. They are followed by the density of comprehensive hospitals, the density of intersections, the density of elementary and middle schools, the proportion of built-up areas, the density of gyms and sports centers, and nighttime light. The importance values of the two most important factors are 8.2 times and 3.6 times higher than the 3rd most important factor “density of comprehensive hospitals”, respectively. The population flow is most influential for the CVIC, followed by the betweenness centrality, the density of elementary and middle schools, nighttime light, compactness of township, the density of intersections, the density of colleges and universities, and the density of comprehensive hospitals. The importance value of the population flow is 3.2 times higher than the 2nd most important factor “betweenness centrality”. Among these top-ranked factors, most are related to urban facilities. There are also transportation-related factors (the density of intersections and betweenness centrality) and activity-related factors (nighttime light and the population flow). Among the 19 variables, compactness of township is ranked in the 5th place for CVIC; however, it is ranked in the 12th place for RCIC.

**Figure 3:**
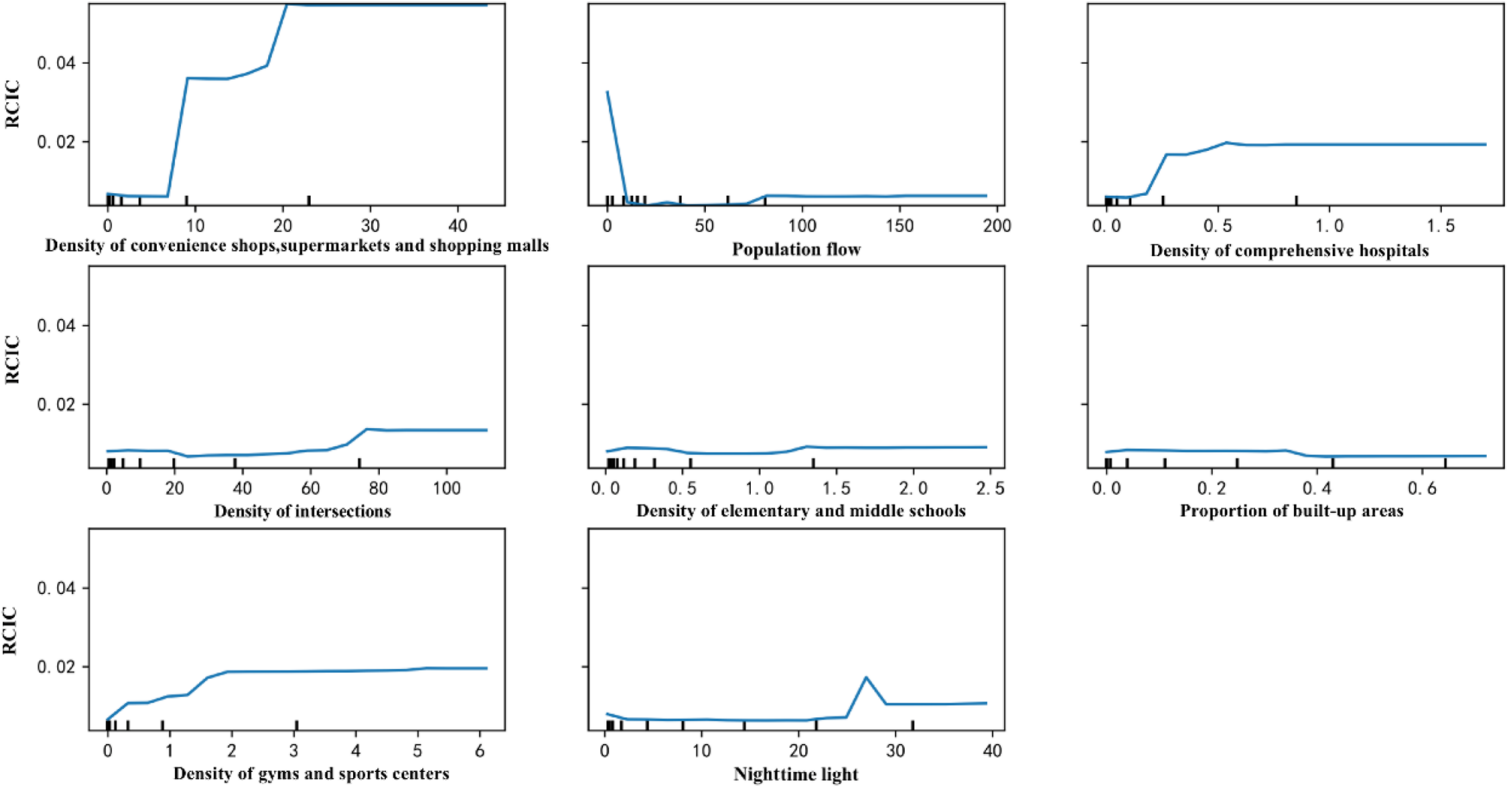
Partial dependence plots between built environment factors and the RCIC. Created using Anaconda Navigator Version 1.9.12 from Anaconda (https://www.anaconda.com).

Looking at the partial dependence plots between the top-eight explanatory variables and RCIC (Fig. 3), it is found that the DoCSS, the density of comprehensive hospitals, the density of intersections, and the density of gyms and sports centers are associated with higher values of RCIC—in other words, more infection cases. Among these influential factors, the DoCSS and the density of comprehensive hospitals show a step-like relationship with the RCIC. For the DoCSS, the density of comprehensive hospitals, the density of intersections, and the density of gyms and sports centers, the thresholds characterizing their maximum impacts on the RCIC are 21/km^2^, 0.7/km^2^, 72/km^2^, and 2/km^2^, respectively, and when the density values are larger than these thresholds, their impacts do not change further. To control the RCIC during the COVID-19 outbreak, it is necessary to pay more attention to townships with a DoCSS higher than 8/km^2^, a density of intersections higher than 70/km^2^, and a density of comprehensive hospitals higher than 0.25/km^2^, through strategies such as rotated opening hours of shopping facilities, road traffic control, and keeping the building environment inside and the surrounding hospitals clean and safe to avoid a sudden leap of infection cases. The RCIC rises with the density of gyms and sports centers when it reaches 0.4/km^2^, implying that in the initial stage of the COVID-19 outbreak, these facilities for discretionary activities can be closed without a serious impact on economic activities. Increases in the density of elementary and middle schools and the proportion of built-up areas are not associated with larger fluctuations of the RCIC; however, its impact becomes smaller when the proportion of built-up areas increases—especially after exceeding 0.3—meaning that the RCIC is higher in rural areas with less built-up areas than urban areas. Accordingly, it is important to take stricter control measures in rural areas with a proportion of built-up areas less than 0.3, where medical facilities and service quality are problematic.

Nighttime light has a positive influence on the RCIC when reaching 25 nanoWatts/cm^2^/sr and shows a strong fluctuation when the value is equal to 25–28 nanoWatts/cm^2^/sr. The nighttime light in 506 townships (occupying 16.9% of the total 2994 townships) are over 25 nanoWatts/cm^2^/sr. Among them, 90 townships are between 25 to 28 nanoWatts/cm^2^/sr; these are widely distributed in relatively developed cities, such as Qingshanlu township in Nanchang, Nangangqu township in Hefei, and Dafeng township in Chengdu. As for the inter-city population flow, it imposes a strong negative impact on the RCIC when it reaches 20%. This indicates that at the beginning of the spread of COVID-19, people living in those townships with very low population flow may pay less attention to protecting themselves and avoiding transmitting the virus to others, while people living in dense townships probably took actions against being infected. For instance, on January 18 in Changtaizhen township in Putian (population flow is 2.5%), there were 24 concentrated infected cases because of an outdoor banquet^17^. However, after reaching 75%, a higher population flow leads to a higher RCIC, and such an impact remains stable if the population flow is larger than 80%. At 194%, the inter-city population flow is highest in the townships of Chongqing, followed by the townships of Nanyang in Henan Province (population flow = 109%) and Yueyang in Hunan Province (population flow = 82%). Other townships in cities with population flows of over 75% are Changsha, Jiujiang, Xinyang, etc. Therefore, the above results support the restriction of inter-city population flow from cities in Hubei Province to these cities.

**Figure 4:**
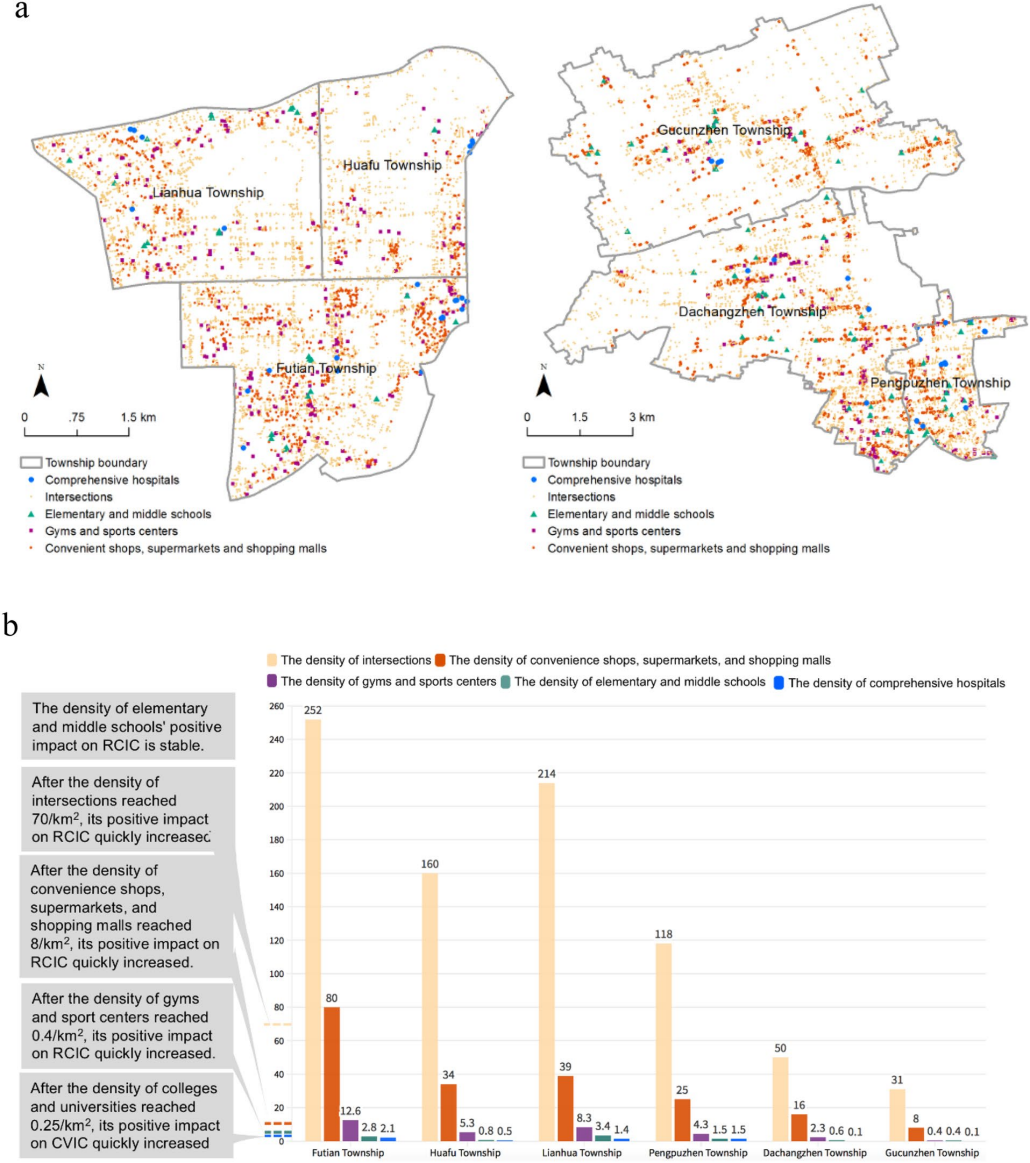
Distributions of major built environment factors at selected townships in Shenzhen and Shanghai. (**a**) Spatial distributions of townships in Shenzhen (left) and Shanghai (right). Created using ArcMap Version 10.2 from ESRI (http://www.arcgis.com/). (**b**) Statistics of density of intersections, the DoCSS, density of gyms and sports centers, density of elementary and middle schools, and density of comprehensive hospitals; the grey boxes refer to the threshold of every density with regard to its impact on the RCIC. Created using Flourish platform (https://flourish.studio).

In order to explain the meaning of the influences of built environment factors in planning practice, we selected several townships in Shenzhen and Shanghai as examples for RCIC (Fig. 4), and several other townships in Beijing and Hangzhou as examples for CVIC. These four cities are first-tier cities in China, with much higher development levels and much larger population sizes than other cities. Shenzhen and Shanghai emphasize economic development, while Beijing and Hangzhou are characterized by traditional Chinese culture. The selection of these four cities for interpreting results about RCIC and CVIC, and the later selection of Beijing and Hangzhou for RCIC and CVIC, have no particular meaning. Other cities can be analyzed in a similar way.

As shown in Fig. 4, the three townships of Futian, Lianhua, and Huafu in Shenzhen cover 10 km^2^, 8 km^2^, and 9 km^2^, respectively, and the three townships of Pengpuzhen, Dachengzhen, and Gucunzhen in Shanghai cover 11 km^2^, 51 km^2^, and 57 km^2^, respectively. The corresponding DoCSS, the density of intersections, the density of comprehensive hospitals, and the density of gyms and sports centers are illustrated in Fig. 4. The DoCSS in the three townships of Shenzhen are 80/km^2^, 39/km^2^, and 34/km^2^, which are all higher than 8/km^2^. In the Gucunzhen township in Shanghai, where only one infected person stayed, the DoCSS is 8/km^2^. Thus, Gucunzhen may be regarded as a good example for planning of convenience shops, supermarkets and shopping malls. In Dachangzhen and Gucunzhen, the values of the density of intersections (50/km^2^ and 31/km^2^) are much lower than the threshold 70/km^2^ (note: there was also only one infected person living in Dachangzhen). This suggests that the built environment related to roads may mitigate a dramatic growth of the RCIC. In Gucunzhen, the density of gyms and sports centers is 0.4/km^2^, which is on the threshold, while in the other five townships the values of the density of gyms and sports centers are all higher than the threshold. The density of comprehensive hospitals in Futian, Huafu, Lianhua, and Pengpuzhen are over 0.25/km^2^. Therefore, more strategies should be considered to keep both the inner environment of comprehensive hospitals and their surroundings safe.

**Figure 5:**
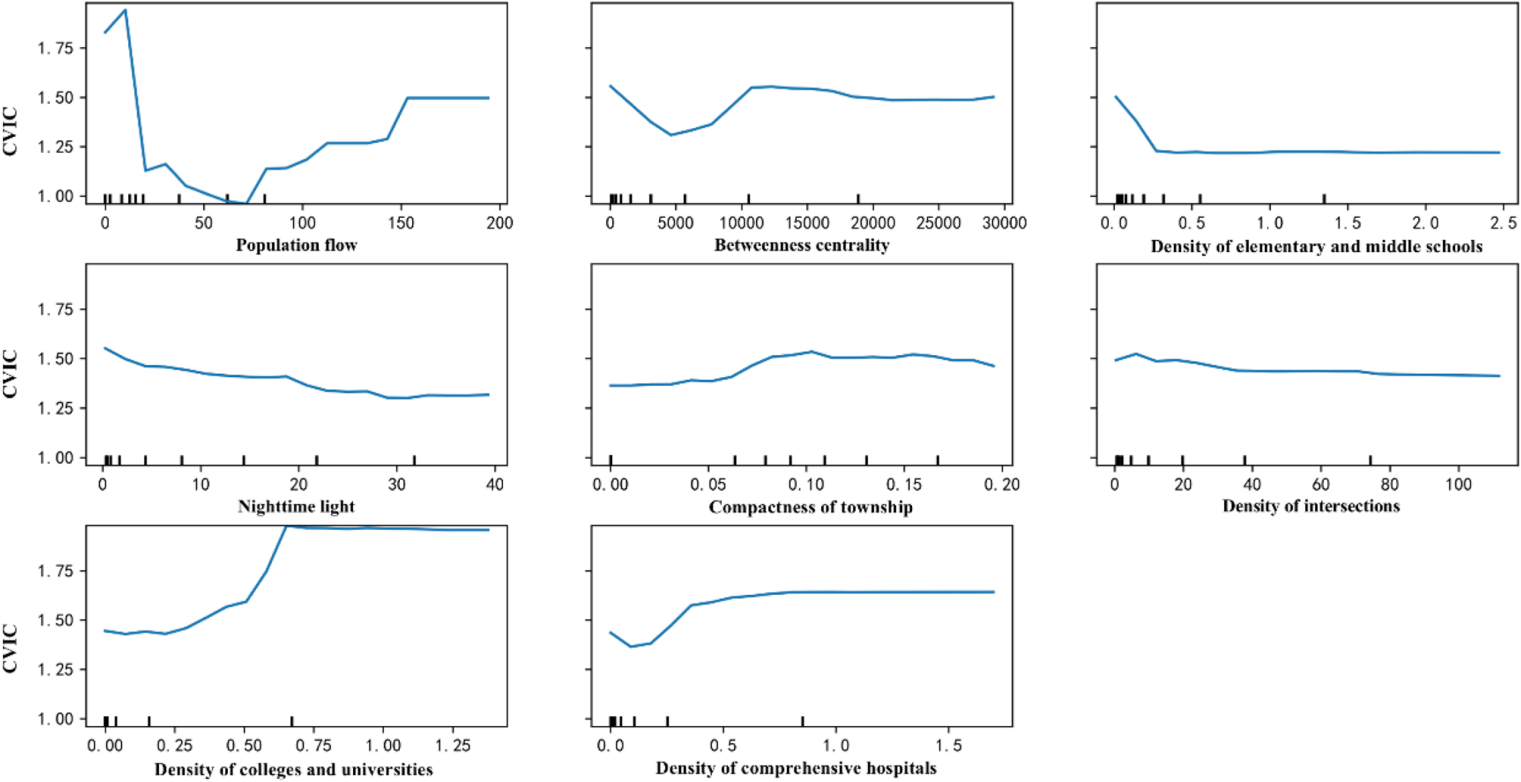
Partial dependence plot between built environment factors and the CVIC. Created using Anaconda Navigator Version 1.9.12 from Anaconda (https://www.anaconda.com).

Focusing on the partial dependence plots between the top-eight explanatory variables and the CVIC (Fig. 5), the density of elementary and middle schools, nighttime light, and the density of intersections have negative impacts on the CVIC; notably, the impact of the density of intersections is positive when it is lower than 5/km^2^. This means the higher the values of the density of elementary and middle schools, nighttime light, and the density of intersections, the lower the fluctuation of their impacts on CVIC; in other words, the better the effects of policy measures in the initial stage of the spread of COVID-19. On the other hand, an opposite association is identified with respect to the density of colleges and universities and the density of comprehensive hospitals, even though the impact of the density of comprehensive hospitals is negative when it is lower than 0.1. When the inter-city population flow, the density of elementary and middle schools, nighttime light, the density of intersections, the density of colleges and universities, and the density of comprehensive hospitals reach 150%, 0.3/km^2^, 30 nanoWatts/cm^2^/sr, 80/km^2^, 0.65/km^2^, and 0.8/km^2^, respectively, their influences become relatively stable as their values increase. Therefore, policy measures should be stricter at townships with the number of colleges and universities per square kilometer being higher than 0.5 or the number of comprehensive hospitals per square kilometer being higher than 0.25. These values are also the thresholds for avoiding a higher RCIC, within which the policy effects in the initial stage are lower.

The impacts of the inter-city population flow and the betweenness centrality on the CVIC greatly fluctuate. More precisely, the CVIC shows a strong negative relationship with the population flow when it is smaller than 75%, but above this percentage, the relationship changes to be positive. Thus, it is necessary to restrict the population flow for enhancing the policy effects at townships with an inter-city population flow larger than 75%; moreover, this is the same as the threshold for avoiding high RCIC. Similarly, the influence of BC on the CVIC first decreases when it is smaller than 5000 and then increases. In this study, the highest BC is 132,451, which is observed in Yuehai township, Shenzhen. The townships with BC around 5000 are Yingbinlu (5020) in Shenyang; Pinghuzhen (5015) in Nantong; Nanpu (5001) and Hongdian (4990) in Wenzhou; and Xiangyang in Fuyang (4981). These cities are classified as medium-sized cities; the population sizes in Shenyang, Nantong, Wenzhou, and Fuyang are 8,320,000, 7,320,000, 9,300,000, and 8,260,000, respectively. It is clear that for the townships where the betweenness centrality values are around 5000, the control measures are more effective than other townships. This may suggest that reopening the economy could be given a higher priority in the townships where the betweenness centrality values are around 5000.

**Figure 6:**
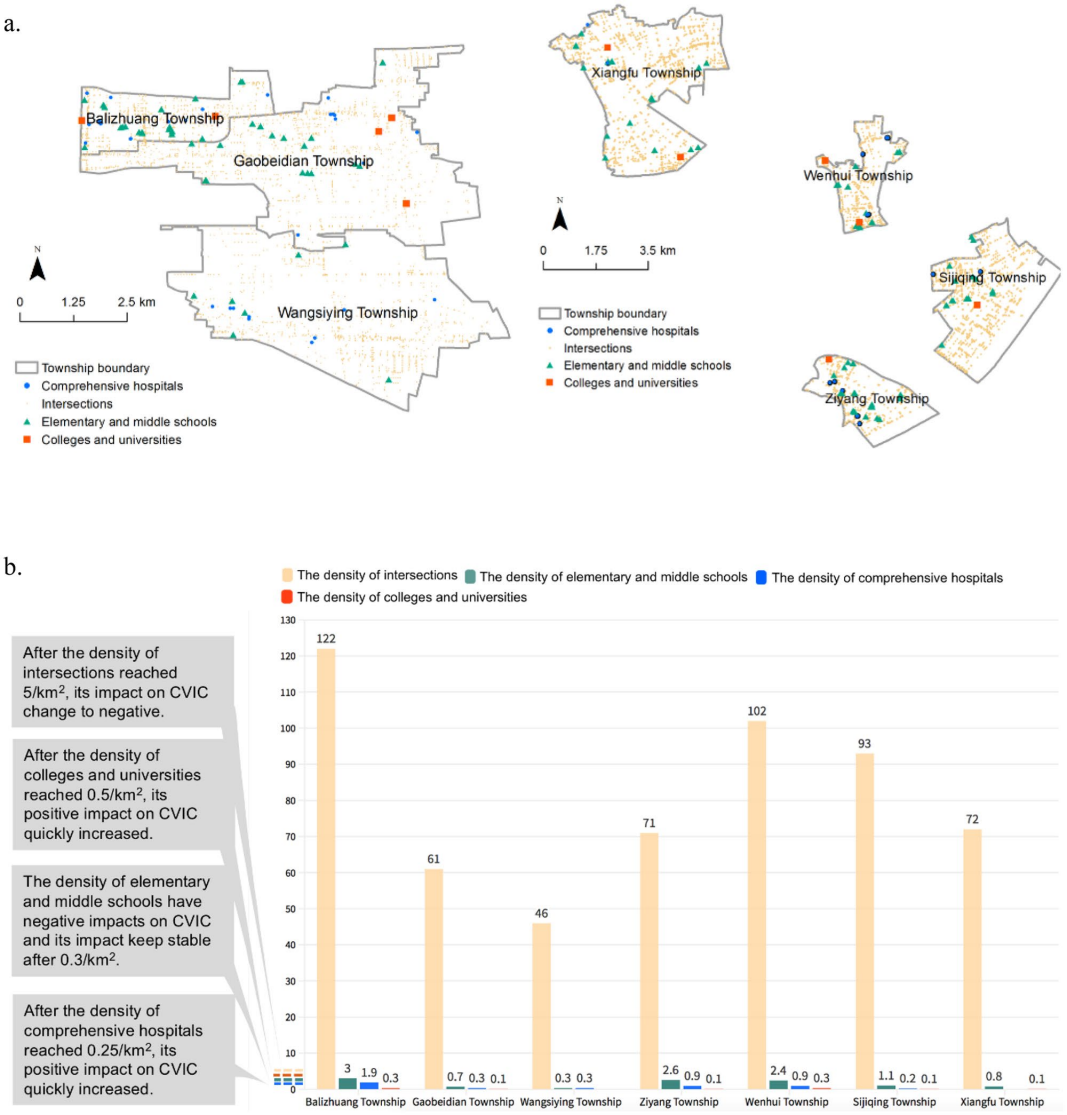
Distributions of major built environment factors to CVIC in selected townships of Beijing and Hangzhou. (**a**) Spatial distributions of townships in Beijing (left) and Hangzhou (right). Created using ArcMap Version 10.2 from ESRI (http://www.arcgis.com/). (**b**) Statistics of density of intersections, density of elementary and middle schools, density of comprehensive hospitals, and density of colleges and universities; the grey boxes refer to the threshold of every density regarding its impact on the CVIC. Created using Flourish platform (https://flourish.studio).

To explain the practical meanings of built environment densities that are important for the CVIC, we selected several townships in Beijing and Hangzhou as examples (Fig. 6). The townships of Balizhuang, Gaobeidian, and Wangsiying in Beijing cover 7 km^2^, 25 km^2^, and 26 km^2^, respectively; the Ziyang, Wenhui, Sijiqing, and Xiangfu townships in Hangzhou cover 8 km^2^, 6 km^2^, 15 km^2^, and 21 km^2^, respectively. In Bailizhuang, Gaobeidian, Ziyang, and Wenhui, the values of the density of comprehensive hospitals are all higher than 0.25/km^2^. Thus, more strategies should be proposed to protect the residents who live near comprehensive hospitals. In all the above townships, the density of colleges and universities is lower than 0.5, suggesting that it is not necessary to control infections in them.

## Discussion

The spread of COVID-19 is associated with spatial agglomerations across China. Clusters with both low RCIC and low CVIC at the same location (Low–Low type) show the largest percentage, followed by the Low–High type. The Low–High type is more dangerous because clusters of low RCIC are surrounded by clusters with high CVIC, which means that policy measures taken in the initial stage may be less effective to prevent/mitigate rapid disease spread at these locations. The DoCSS and the inter-city population flow were the two most important variables for the RCIC, whereas the population flow was the most important variable to measure the policy effects (i.e., the CVIC). When the DoCSS, the density of intersections, and the density of gyms and sports centers reach 21/km^2^, 72/km^2^, and 2/km^2^, respectively, positive associations of these density indicators with the RCIC become maximal and remain constant as the indicators further increase. Only after the population flow exceeds 75% does it start showing a positive relationship with RCIC; this relationship remains stable when the population flow gets to 80%. For policy effects, the density of colleges and universities and the density of comprehensive hospitals had negative impacts on policy effects in the initial stage of the COVID-19 outbreak. Notably, when the density of comprehensive hospitals was at less than 0.1/km^2^, it had a small positive trend. Accordingly, it was observed that increasing the betweenness centrality associated with increasing policy effects in the initial stage of COVID-19 spread before the value of 5000, and a reversed trend was observed after 5000.

Based on the above evidence, our findings suggest that infection cases may be suppressed if the DoCSS could be kept within 8/km^2^. Furthermore, gyms and sports centers could be closed for a longer time, special attention could be paid more to townships with a density of comprehensive hospitals over 0.25/km^2^, and the density of intersections could be kept within 70/km^2^. For the townships with an inter-city population flow of less than 75%, the inter-city travel restrictions (from Hubei Province) may not be made compulsory. In addition, all elementary and middle schools in the same township may be closed or reopened at the same time. In order to make policy measures more effective, the resumption for townships with a density of colleges and universities larger than 0.5/km^2^ should be restricted, while the resumption of the townships with a betweenness centrality value around 5000 should be given higher priority. Strategies that address the impacts of COVID-19 with respect to comprehensive hospitals and the inter-city population flow could be made in a similar manner.

Our study contributes to the growing body of scholarship on COVID-19 and the built environment by examining the associations between the spread of COVID-19 and the built environment factors that indirectly capture “inferred” human mobility, rather than “observed” human mobility. It presents the spatial agglomerations of RCIC and CVIC observed before the lockdown throughout China, where the latter represents the policy effects in the initial stage of the spread of COVID-19. We adopted the random forest approach to objectively identify influential built environment factors and other factors. In particular, we found that the influences are nonlinear and each has an obvious threshold value. Such thresholds provide objective evidence on how to control the spread of COVID-19 and are also insightful for future urban planning against pandemics.

The findings—especially the various thresholds—from this study present practical insights for both immediate and recovery measures as well as long-term policymaking about how to control and manage the spread of COVID-19. As for immediate and recovery measures, the findings suggest that it is not necessary to implement the same measures across areas in a uniform manner; this can lead to unnecessary damage to the economy and society. Immediate and recovery measures should be taken in a flexible way reflecting various differences across locations, types of activities, population groups, etc., in order to adapt to the new normal brought by COVID-19. From a long-term perspective, the findings provide hints about how to redesign the built environment and physical distancing in various contexts. Physical distancing design refers to not only within-building space design but also the design of communities, cities, regions, and whole nations. To enhance the resilience of society with regard to future pandemics, it is worth conducting cross-country comparative research reflecting cultural and geographical differences as well as socioeconomic heterogeneities.

## Methods

### Data sources and specifications of built environment factors

We selected 19 explanatory variables, including built environment factors (related to urban facilities, land use, and transportation infrastructure), the level of nighttime activities, and the inter-city population flow (from Hubei Province) (for details, refer to Supplementary Table 1). Urban facilities represent places where people perform their daily activities via face-to-face communications, including facilities of daily life services (e.g., supermarkets and pharmacies), facilities of physical activities (e.g., gyms and sports centers), social communication facilities (e.g., restaurants, cafes and bars), medical facilities (e.g., hospital and clinic), education facilities (e.g., schools and libraries), and leisure and recreation areas (e.g., parks, squares, and other green open spaces). Close contacts among people in built-up areas, which represent a physical environment that supports human activities and communications, are much higher than in rural areas^18,19^. Nighttime light records the intensity of light at Earth’s surface at night, thereby reflecting the magnitude of nighttime activities. Outdoor spaces are known to be important factors to improve chronic physical and mental diseases^20,21^, partly because of the face-to-face communications with other persons which take place in outdoor environments. Considering that face-to-face contacts are the main channel to spread the COVID-19 virus, it is realistic to assume that the greater the number of facilities allowing face-to-face communications, the higher the probability of spreading the virus or being infected by the virus. Thus, we considered land use density and selected eleven variables to capture social contacts via urban facilities within built-up areas. On the other hand, even though transportation vehicles and facilities are not the main infection cluster (e.g., only 1.2% of the 2830 clusters found in France were transport), infections due to the use of transport were likely at early stages of the pandemic (e.g., in Sweden, the infection risk of taxi drivers was 4.8 times higher than all other occupations, followed by bus and tram drivers; in the UK, the mortality rates of male taxi drivers, chauffeurs, and bus and coach drivers were 1.6 times higher than the general male population)^22^. To reflect the influence of transportation, we selected three factors: the betweenness centrality, the density of road intersections, and the density of bus and subway stations. The betweenness centrality of a location, measured in terms of the times of the shortest routes in the entire road network passed through the location, is identified to be an imperative factor in understanding the dynamics of the spread of infectious diseases via direct person-to-person transmission^23^. The density of road intersections and the density of bus and subway stations reflect the accessibility to places, facilitating face-to-face interactions^24^. The population flow is especially important in the context of the outbreak of COVID-19. For instance, it is argued that the spread of COVID-19 in China was associated with the massive migration from Wuhan during the Spring Festival season^25^. Therefore, we selected the inter-city population flow from Hubei Province to a township of interest as a factor in this study.

- *Township-level administrative boundary* We adopted the township-level administrative boundary as the basic study unit. Among the total 39,007 townships in China, there were 2994 townships with data on the number of infection cases (2108 townships had infection cases and infected people stayed in 886 townships) (Supplementary Fig. 1). Data from these 2994 townships are used in this study.
- *Points of interest (POIs) in China in 2018* A POI refers to a location that a person may find useful or interesting. We collected data of POIs in China from Amap (https://lbs.amap.com), which included a total of 44,913,302 POIs in 2018 (Supplementary Fig. 2). Amap is one of the largest mapping service application and technology companies in China, offering satellite imagery, street maps and location-based services. There are 23 types of POIs that are used to measure the following variables: the DoCSS, the density of restaurants and cafes, the density of gyms and sports centers, the density of pharmacies, the density of comprehensive hospitals, the density of clinics and health centers, the density of elementary and middle schools, the density of colleges and universities, the density of subways or bus stops, and the degree of land use diversity by computing the information entropy of POIs in each township. Residential areas were also identified to calculate the travel time from a residential area to its nearest hospital.
- *Road network in China in 2018* We obtained road network data from Amap in 2018. Because the original streets contain two-way lanes or overpasses, we simplified the roads by following an existing method^26^. As a result, a total 1,589,644 processed roads were used to calculate the betweenness centrality and the density of intersections (Supplementary Fig. 2).
- *Public space in 2018* Various public spaces such as parks, squares, and other green open spaces are included. We extracted relevant data by color from Amap and checked POIs in order to calculate the proportion of these areas (public space) as a built environment factor.
- *Built-up areas in China in 2015* We collected data of built-up areas in China in 2015, with a 30 m resolution from the Resource and Environment Data Cloud Platform (http://www.resdc.cn/data.aspx?DATAID=184) to calculate the density of built-up areas and their land patterns. This was done to measure whether a built-up area is more compact or more disperse for each township (Supplementary Fig. 2).
- *Inter-township connections* We categorized townships into three types: strong connection, medium connection, and low connection. We accomplished this by referring to an inter-township connections degree^27^.
- *Nighttime light in China in 2018* For every township, we collected the monthly average nighttime light in China in 2018 from the NPP-VIIRS satellite from Google Earth. This data has been shown to be more reliable than traditional data for spatializing socioeconomic indicators such as economic activities, energy consumption, and population^28,29^. After filtering outliers and removing background noise, we used the nighttime light to reflect the nighttime activities.
- *Travel time to the nearest hospital* We collected data concerning the travel time from a residential area to its nearest hospital through the path planning interface on Baidu direction lite API (http://lbsyun.baidu.com/index.php?title=webapi/direction-api-v2). We then calculated the average time for all residential areas in every township as a variable representing the built environment.
- *Population flow* This data came from Baidu Migration Production (http://qianxi.baidu.com/). In every city, we computed the cumulative outflow population percentage, which is a cumulative value of the daily proportion of the outflow population to the city of total outflow population from Hubei Province between January 1 to January 27 (note: all cities in Hubei Province were locked down on January 27). The townships that are in the same city are given the same percentage.

### Data of the spread of COVID-19

We collected the numbers of township-level infection cases of COVID-19 that were observed on February 13 via the GeoHey platform from Tencent and the People's Daily (https://gitee.com/geohey/gh-2019-nCoV-community-data). First, the RCIC is calculated as the number of diagnosed infection cases which is then divided by the population data from the Sixth National Population Census of the People's Republic of China^30^. Second, the CVIC was computed over the period between February 6 and 13, 2020. Moreover, the CVIC can be used to evaluate the effectiveness of policy^31^. The CVIC reflects the fluctuations of infection cases and can be used to measure the effects of policies to mitigate the spread of COVID-19 in the initial stage. A higher CVIC reflects a lower effectiveness of the earlier policies of COVID-19 spread in a township—and vice versa.

In the initial stage in China, before Wuhan was locked down on January 23, 2020, the Chinese government implemented strict policy measures such as the closure of the South China Seafood Market, and disclosed scientifically-supported risk information about COVID-19 in order to maintain social stability and avoid social panic. After January 23, the government encouraged people to stay at home, discouraged mass gatherings, canceled or postponed large public events, conducted body temperature detection, stopped the operation of public transportation, and closed schools, universities, and cinemas^32,33^. Furthermore, the whole country was locked down from January 30. Further details on data sources and data processing are available in the Supplementary Information.

### Bivariate local indicators of spatial association (BiLISA)

We measured the spread of COVID-19 in two dimensions: the RCIC and CVIC (i.e., policy effects in the initial stage). We applied the BiLISA-based method in the GeoDa software (https://geodacenter.github.io) and set the threshold of significance at 0.1 to detect the local relationship between the above two variables by calculating the following BiLISA ($I_{i,kl}$) (details refer to Anselin^34^).

1 $$I_{i,kl} = Z_{i,k} \mathop \sum \limits_{j = 1}^{N} w_{ij} Z_{j,l} ,$$

2 $$Z_{i,k} = \left( {X_{i,k} - X_{k,mean} } \right)/\sigma_{k} ,$$

3 $$Z_{j,l} = \left( {X_{j,l} - X_{l,mean} } \right)/\sigma_{l} .$$

Here, *N* refers to the number of spatial units; $X_{i,k}$ and $X_{j,l}$ indicates the values of variable $k$ in spatial unit $i$ with variable $l$ in its neighboring spatial units $j$ ($j \ne i$); $X_{k,mean} { }$ and $X_{l,mean}$ represent the average values of the variables $k$ and ${ }l$, respectively; $\sigma_{k}$ and $\sigma_{l}$ means the variances of variables $k$ and ${ }l$, respectively; and $w_{ij}$ measures the spatial weight between spatial units *i* and *j*. A conditional randomization approach is used to evaluate the significance of $I_{i,kl}$ in three steps: (1) the variable values are randomly permuted over the locations; (2) the local Moran’s I are calculated for the permuted dataset; and the resulting distributions are used to evaluate the significance of the observed local spatial autocorrelation against the random pattern^35^. The local neighbors $j$ are identified by their proximity to area $i$ in straight-line distance, and their values are used for calculating the $I_{i,kl}$ for area $i$. The sizes of townships in China between the western and eastern areas presented quite a difference. When defining the local neighbors, we used a fixed number of local neighbors for each location to avoid bias.

### Random forest approach

In order to analyze influential factors to the spread of COVID-19, we adopted a random forest approach. It is an efficient and effective machine learning method that builds a large number of decision trees, each of which is regressed with respect to a randomly selected subset of training samples^36,37^. Modeling results are validated using data beyond the training samples. Thus, cross-validation using external data is not required. The validation samples are also used to measure the relative importance of each variable^38^. The outputs from all of the trees are then averaged, providing predictive accuracy^36^. For learning algorithms and tuning parameters, the default values of these parameters work very well in practice. Details for all parameters are available in the Supplementary Information.

The random forest approach is used because of the following advantages compared to other models. First, it can automatically detect various nonlinear associations of a very large set of predictors (or explanatory variables) with a target variable (or dependent variable). Second, the collinearity issue^39^ afflicting all regression analysis approaches does not matter to the random approach. Third, it can automatically identify thresholds that determine the nonlinear associations. Fourth, all three of the above features are supported by its tree-based robust estimation and reliable validation mechanisms. These attractive features are not shared by the popular multiple linear regression model, logistic regression model, or more advanced general linear model. Furthermore, the random forest approach can avoid the overfitting issue when the sample size is very large, as it is in this study. Unlike these existing models, the random forest approach evaluates the association and statistical significance of each predictor by calculating its relative importance, which is measured by IncMSE: the higher the value of IncMSE, the more important the corresponding factor^40,41^. For the random forest approach, its classification accuracy is high, operation speed is fast, operation results are robust, and generalization ability is strong^36^. The random forest approach was therefore chosen to reveal the key built environment factors affecting the spread of COVID-19 (i.e., RCIC/CVIC).

Because of these features, it is not necessary to sort out suitable factors in advance, for example, by addressing the collinearity issue. Neither is it necessary to limit the number of predictors, in principle. Therefore, we first obtained as many potential factors as possible, as explained in the data section above, and then directly introduced those factors to estimate their influences on RCIC and CVIC, respectively, using the random forest approach. After that, we ranked all 19 built environment factors by the relative importance of each factor. Then we examined the factors that are significantly associated with the spread of COVID-19 by using partial dependence plots. Partial dependence plots depict the functional relationship between a small number of input variables and a dependence variable, and show how the dependence variable partially depends on the values of the input variables of interest. Details of the partial dependence plots are given in the Supplementary Information.

## Summary

Based on multisource big data, this nationwide study based on a random forest approach presents various types of objective evidence showing that the built environment is associated with the township-level spread of COVID-19 in China. The main findings can be summarized as follows. First, as for the ratio of cumulative infection cases (RCIC), it is mostly affected by the density of convenience shops, supermarkets and shopping malls (DoCSS) and the inter-city population flow from Hubei Province. These two factors are far more important than other factors. Concerning the coefficient of variation of infection cases (CVIC), it is mostly influenced by the population flow, which is 3.2 times more important than the 2nd most important factor. Second, spatial agglomerations of RCIC and CVIC revealed that the Low–Low type is dominant in the townships of China. Third, higher RCIC are linked with higher values of the DoCSS, the density of comprehensive hospitals, the density of intersections, and the density of gyms and sports centers. The impacts of the DoCSS, the density of comprehensive hospitals, the density of intersections, and the density of gyms and sports centers on RCIC become the largest when these density values are higher than 21/km^2^, 0.7/km^2^, 72/km^2^, and 2/km^2^, respectively, and maintain unchanged as the density values further increase. Fourth, higher values of the density of elementary and middle schools, nighttime light, and the density of intersections lead to a lower CVIC, implying better policy effects. In order to effectively suppress the spread of COVID-19, stricter policy measures are required at townships with a certain density of colleges/universities (higher than 0.5/km^2^) or comprehensive hospitals (higher than 0.25/km^2^). Thus, nonlinear effects of built environment factors on the spread of COVID-19 are revealed, which all have obvious thresholds. Such revealed thresholds are useful to guide future density-driven urban planning for preventing future pandemics.

## Supplementary Information

Supplementary Information.

## Data Availability

All data supporting the findings of this study are available within the article and its Supplementary Information.
