# Supplementary material for: Diverse and nonlinear influences of built environment factors on COVID-19 spread across townships in China at its initial stage
Source: Sci Rep. 2021 Jun 14;11:12415. doi: 10.1038/s41598-021-91849-1 (PMC8203673; doi:10.1038/s41598-021-91849-1)
Supplement: Supplementary file 1 — Supplementary Information. [file 41598_2021_91849_MOESM1_ESM.docx]

**Supplementary Information for:**

**Diverse and nonlinear influences of built environment factors on COVID-19 spread across townships in China at its initial stage**

**Authors: Shuang Ma ^1#^, Shuangjin Li ^2#^, Junyi Zhang ^2,3*^**

**Affiliations:**

^1^ Research Center for Advanced Science and Technology, The University of Tokyo, Tokyo, 153-8904, Japan

^2^ Graduate School for International Development and Cooperation, Hiroshima University, Higashi Hiroshima, 739-8529, Japan

^3^ Graduate School of Advanced Science and Engineering, Hiroshima University, Higashi Hiroshima, 739-8529, Japan

*^#^ Joint first authors: these authors contributed equally to the work*

**Corresponding author: zjy@hiroshima-u.ac.jp*

Methods: Data sources and processing

**Township-level administrative boundaries in China**

In China, there are three types of township-level administrative units (i.e., townships in this study): *Jiedao* (sub-district), *Zhen* (town), and *Xiang* (township). *Jiedao* and *Zhen* are administrative units in urban areas and *Xiang* is an administrative unit in a rural area.

**Points of interest (POIs) in China in 2018**

To measure the land use diversity at the township level, we calculated the degree of mixed land use based on the concept of information entropy with respect to the following 15 types of POIs: “residential community, community services, companies, office buildings, financial services, legal services, governmental institutions, educational institutions, medical institutions, commercial sites, catering sites, entertainment, hotels, tourism, transport facilities, and other facilities”.^[[1]](#endnote-2)^ These POIs reflect “live, work, and play”.

$M=-\sum_{i=1}^{n} p_{i}lnp_{i}$ (s1)

where $M$ indicates the degree of land use diversity of a township, $n$ refers to the number of POI types in a township, and $p_{i}$ represents the proportion of a specific POI type within the township. We define the mixed functions as a variable reflecting the urban facilities.

**Road networks in China in 2018**

In line with Long and Liu^[[2]](#endnote-3)^, road networks in China were preprocessed by merging divided streets using the “merge divided roads” tool, simplifying the street network using the “thin road network” tool, and checking the topology.

**Public space in 2018**

To collect this data, we first extracted parks, squares and other green open spaces by color in Amap (<https://lbs.amap.com>) and transferred the area data to the vector data in ArcGIS (Version 10.2 from ESRI: http://www.arcgis.com/). In order to check the accuracy of the public space data, POIs are also used here.

**Built-up areas in China in 2015**

This data is available at: <http://www.resdc.cn/data.aspx?DATAID=184>. Land use patterns are captured by the compactness index (CI), which is calculated by the following equation:

$CI=\frac{P}{2\times\sqrt{A\times\pi}}$ (s2)

where $P$is the perimeter of built-up area in a township, $A$ is the area of built-up area in the township. The higher the $CI$, the more compact the built-up area in the township; the lower the $CI$, the built-up area is more dispersed.

**Inter-township connections**

We categorized townships into three types to represent inter-township connections: strong connection, medium connection, and low connection. The aforementioned categorization is in line with a previous study on urban functional areas in China at the township level based on commuting car-hailing records.^[[3]](#endnote-4)^

**NPP-VIIRS nighttime light in China in 2018**

We collected monthly averaged radiance composite images for every township in 2018 from the VIIRS Stray Light Corrected Nighttime Day/Night Band (DNB) Composites Version 1 dataset from Google Earth Engineer (GEE) (available at: <https://ngdc.noaa.gov/eog/viirs/download_dnb_composites.html>). This data has a procedure to correct for stray light, according to Mills, Weiss, and Liang, to remove almost all of the haze and striping, and improves the dynamic range by two orders-of-magnitude: as a result, it went as low as 1×10-10 Wcm-2 sr-1.^[[4]](#endnote-5)^ The preprocessing tasks include two steps: filtering of extreme values and the removal of background noise surface. Extreme values exist because temporal lights and fires are not filtered in NTL. Shanghai is the most advanced economic center in China and the pixel value cannot exceed the highest value of night light data in Shanghai. Thus, we used the highest radiance value of Shanghai for every month of 2018 as a threshold to correct outliers. We deleted the pixels with a radiance value exceeding the monthly thresholds in Shanghai and calculated the average NTL for every township. To deal with the background noise, we set the pixel with a negative value to 0 so as to remove noise from the data.

**Travel time to the nearest hospital**

The real travel time from a residential area to its nearest hospital by public transport is calculated through a path planning interface on Baidu direction lite API (more information about this API is available at: <http://lbsyun.baidu.com/index.php?title=webapi/directionlite-v1>). This path planning interface has high accuracy and strong authenticity, and can accurately reflect certain details such as walking time while taking public transportation, and passengers’ transfer time between two rail stations. In this study, we selected a period between four to five o'clock in the afternoon on working days to evaluate the time costing. The residential area and the hospital are extracted by POIs. Furthermore, in order to distinguish the nearest hospitals of the residential area, the “near analysis” in ArcGIS was utilized.

**Data of the spread of COVID-19**

The number of COVID-19 diagnoses is available at <https://gitee.com/geohey/gh-2019-nCoV-community-data/tree/master/data>. To get the cumulative number of infection cases at a township level, the spatial join tool in ArcGIS is used. According to the Ministry of Transport of the People’s Republic of China, Chinese cities were “shut off”from public transportation facilities on January 30, 2020. To reflect the 14-day incubation period, the cumulative number of infection cases is used until February 13, 2020. We also collected the population data of the Sixth National Census which is the newest census so far at the township level (available at: <https://www.beijingcitylab.com/data-released/data1-20/>). The equation for calculating the ratio of cumulative infection cases (RCIC) is as follows:

${cor}_{i}=\frac{c_{i}}{p_{i}}$ (s3)

where ${cor}_{i}$ is the RCIC in township *i*, $c_{i}$is the number of cumulative infection cases in township *i*; $p_{i}$ is the number of population in township *i*.

We calculated the daily number of infection cases at the township level from February 6, 2020 (the data at community level is available from this day) to February 13, 2020, and used the coefficient of variation of infection cases (CVIC) to evaluate the policy effect as follows:

${CV}_{D,i}=\frac{\sigma_{D,i}}{{Mean}_{D,i}}$ (s4)

where ${CV}_{D,i}$ is the CVIC in township *i*, $\sigma_{D,i}$is the standard deviation of the daily number of new infection cases in township *i*, and ${Mean}_{D,i}$ is the mean of the aforementioned daily number. ${CV}_{D,i}$explains the fluctuation (increasing/reducing) degree of infection cases.

Method: Random forest approach – parameter design

First, the number of trees was set to 500, which is the default value in the package RandomForestRegressor. In principle, the number of trees should be as large as possible. In practice, however, a few hundred trees are usually sufficient to reach the best performance.^[[5]](#endnote-6)^ Second, the maximum depth of the trees was set to 31. Third, 80% of the original data were used for training and 20% for testing. Fourth, the maximum features parameter was set to “auto”.

Method: Random forest approach – partial dependence plots

Partial dependence plots (PDPs) are useful to uncover any black box machine learning model.^7^ Generally speaking, a regression or classification function, *f*, is dependent on various explanatory variables or predictors. Define $f\left( X \right)=f\left( X_{1}, X_{2},X_{3},\ldots X_{s} \right),$where$X=(X_{1}, X_{2},X_{3},\ldots X_{s})$ are the predictors. The partial dependence of $f$ on a predictor $X_{j}$ is the expectation of $f$ with respect to all the predictors except $X_{j}$. Let $X(-j)$ indicate all the variables except $X_{j}$. Then, “$f_{j}\left( X_{j} \right)=E_{X\left( -j \right)}[f\left( X \right)]$” is the equation of the partial dependence of $f$ on $X_{j}$. In practice, this expectation is estimated by fixing the values of $X_{j}$, and averaging the prediction function over all the combinations of the other (observed) predictors in the data set. With this process, prediction from the entire dataset is required for each value of $X_{j}$ in the training data.^[[6]](#endnote-7)^ In this research, PDPs visualize how each predictor variable influences the predicted RCIC/CVIC using the “sklearn.inspection package” in Python. The X-axis value of a partial dependence plot is obtained by selecting a sequence of values between the minimum and maximum of each variable. The Y-axis value of a partial dependence plot is determined by averaging all possible model predictions with the dataset when the value of the objective predictor is X.


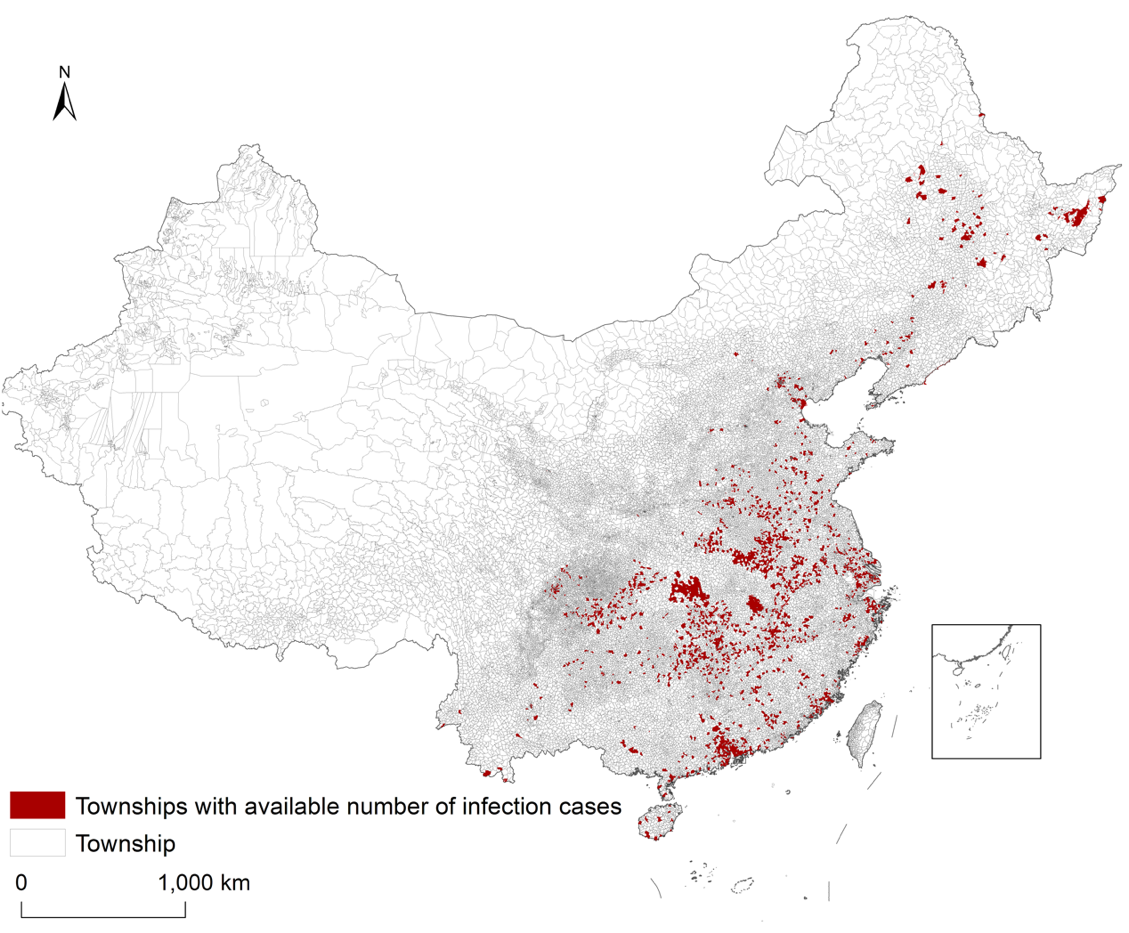


Created using ArcMap Version 10.2 from ESRI (http://[www.arcgis.com/](http://www.arcgis.com/)).

Supplemental Figure 1. Township-level administrative boundaries in China


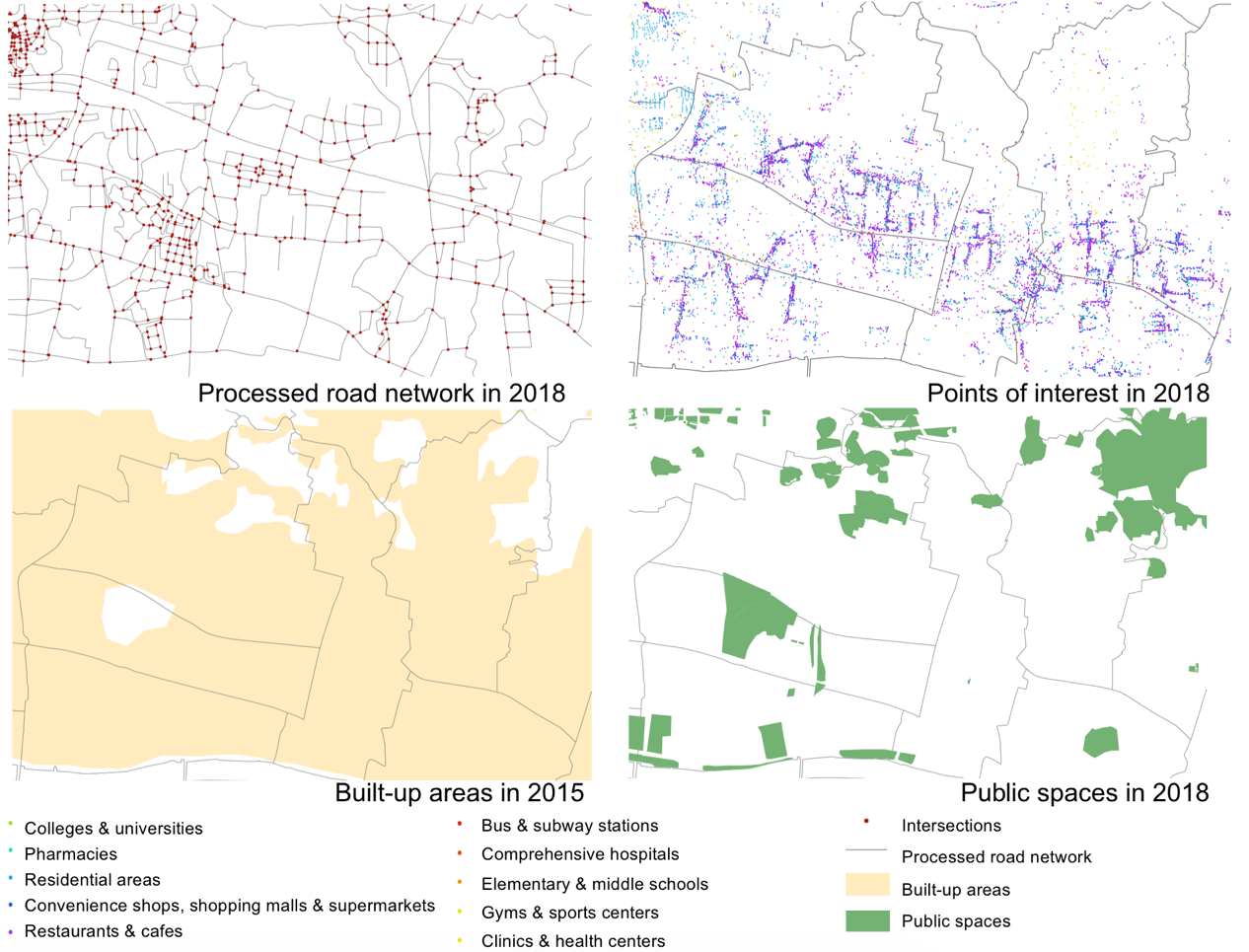


Created using ArcMap Version 10.2 from ESRI (http://[www.arcgis.com/](http://www.arcgis.com/)).

Supplemental Figure 2. Typical data used in this study (processed road networks in 2018, POIs in 2018, built-up areas in 2015, and public spaces in 2018, taken from a sample in China)

Supplemental Table 1. List of explanatory variables for describing the spread of COVID-19

| Dimensions | Aspects | Explanatory variables used in this study | Data sources |
| --- | --- | --- | --- |
| Urban facilities | Daily life services | Degree of land use diversity | Points of interest (POIs) from Amap (available at: https://lbs.amap.com/api/webservice/guide/api/search) |
|  |  | Density of restaurants and cafes | POIs from Amap (available at: https://lbs.amap.com/api/webservice/guide/api/search) |
|  |  | Density of convenience shops, supermarkets and shopping malls (DoCSS) | POIs from Amap (available at: https://lbs.amap.com/api/webservice/guide/api/search) |
|  |  | Density of pharmacies | POIs from Amap (available at: https://lbs.amap.com/api/webservice/guide/api/search) |
|  | Physical activities and social communications | Proportion of parks, squares and other green open spaces | Extract parks, squares and other green open spaces by color in Amap and transfer the area data to vector data in ArcGIS. In order to check the accuracy of the data, POIs are also used here. |
|  |  | Density of gyms and sports centers | POIs from Amap (available at: https://lbs.amap.com/api/webservice/guide/api/search) |
|  | Medical facilities | Density of comprehensive hospitals (the AAA class and other comprehensive hospitals) | POIs from Amap (available at: https://lbs.amap.com/api/webservice/guide/api/search) |
|  |  | Density of clinics and health centers | POIs from Amap (available at: https://lbs.amap.com/api/webservice/guide/api/search) |
|  |  | Travel time from a residential area to its nearest hospital | The actual travel time from a residential area to the nearest hospital by public transport is calculated through path planning interface on Baidu direction lite API (more information about this API is available at: <http://lbsyun.baidu.com/index.php?title=webapi/directionlite-v1>). Residential areas and hospitals are extracted by POIs, and in order to distinguish the nearest hospital of a residential area, the “near analysis” in ArcGIS was applied. |
|  | Education facilities | Density of elementary and middle schools | POIs from Amap (available at: https://lbs.amap.com/api/webservice/guide/api/search) |
|  |  | Density of colleges and universities | POIs from Amap (available at: https://lbs.amap.com/api/webservice/guide/api/search) |
| Transportation infrastructure | Centrality | Betweenness centrality | Road networks from Amap (available at: https://ditu.amap.com) |
|  | Accessibility | Density of road intersections | Road networks from Amap (available at: https://ditu.amap.com) |
|  |  | Density of bus and subway stations | POIs from Amap (available at: https://lbs.amap.com/api/webservice/guide/api/search) |
| Land use | Built-up areas | Proportion of built-up areas | Resource and Environment Data Cloud Platform (available at: <http://www.resdc.cn/data.aspx?DATAID=184>). |
|  |  | Compactness of township | See equation (s2) |
|  | Inter-township connections | Functional urban areas based on Didi car-hailing records | According to a previous study of urban function areas in China at the township level based on commuting car-hailing records.^3^ |
| Social-economic parameter | Nighttime activities | Nighttime light | Monthly averaged radiance composite images from VIIRS Stray Light Corrected Nighttime Day/Night Band (DNB) Composites Version 1 dataset from Google Earth Engineer (GEE) (available at: <https://ngdc.noaa.gov/eog/viirs/download_dnb_composites.html>) in 2019. |
| Population flow | Population flow | Inter-city population flow (total percentage of migration from Hubei Province) | This is from Baidu Migration Production, based on its location-based service (available at: http://qianxi.baidu.com). We calculated the total percentage of population flow from Hubei Province from January 01 to January 27 at the city level and gave the townships in the same city the same percentages. |
| **Epidemic conditions** | | | |
| Ratio of cumulative infection cases (RCIC) | | | See equation (s3) |
| Coefficient of variation of infection cases (CVIC) | | | See equation (s4) |

**Reference**

1. Li, J., Long, Y. & Dang, A. Live-work-play centers of Chinese cities: identification and temporal evolution with emerging data. *Comput Environ Urba*. **71**, 58-66 (2018). [↑](#endnote-ref-2)
2. Long, Y. & Liu, L. How green are the streets? An analysis for central areas of Chinese cities using Tencent Street View. *Plos One* **12**, 1-18 (2017). [↑](#endnote-ref-3)
3. Ma, S. & Long, Y. Functional urban area delineations of cities on the Chinese mainland using massive Didi ride-hailing records. *Cities* **97**, 102532-102545 (2020). [↑](#endnote-ref-4)
4. Mills, S., Weiss, S. & Liang, C. VIIRS day/night band (DNB) stray light characterization and correction. *Proc. SPIE* **8866,** Earth Observing Systems XVIII, 88661P (2013). [↑](#endnote-ref-5)
5. Couronné, R., Probst, P. & Boulesteix, A.L. Random forest versus logistic regression: a large-scale benchmark experiment. *BMC Bioinformatics***19**, 270 (2018). [↑](#endnote-ref-6)
6. Cutler, D.R. et al. Random forests for classification in ecology. *Ecology* **88,** 2783-2792 (2007). [↑](#endnote-ref-7)
